# What Mediates the Relationship Between Ethnic Discrimination and Stress? Coping Strategies and Perceived Social Support of Russian Immigrants in Germany

**DOI:** 10.3389/fpsyt.2020.557148

**Published:** 2020-09-15

**Authors:** Andreas Goreis, Frank Asbrock, Urs M. Nater, Ricarda Mewes

**Affiliations:** ^1^ Department of Clinical and Health Psychology, Faculty of Psychology, University of Vienna, Vienna, Austria; ^2^ Outpatient Unit for Research, Teaching and Practice, Faculty of Psychology, University of Vienna, Vienna, Austria; ^3^ Department of Psychology, Chemnitz University of Technology, Chemnitz, Germany

**Keywords:** ethnic discrimination, stress, Russian immigrants, coping, social support

## Abstract

**Objective:**

Experiences of ethnic discrimination may constitute major stressors for ethnic minority groups. This study examined the associations between different forms of ethnic discrimination and levels of perceived stress in Russian immigrants living in Germany, taking into account potential moderating (in-group identification) and mediating (coping and social support) factors.

**Methods:**

Russian immigrants (*N* = 308) were assessed using online questionnaires (e.g., perceived stress scale, behaviors from intergroup affect and stereotype treatment scale, and brief COPE). Three forms of ethnic discrimination were examined: active harm (e.g., open aggression), passive harm (e.g., paternalistic behavior), and everyday discrimination (e.g., receiving poor service). Moderation by in-group identification and mediation *via* coping and social support were tested.

**Results:**

Passive harm was more prevalent than everyday discrimination and active harm. Passive harm and everyday discrimination were associated with higher perceived stress (*r*s = .22 and .18, *p*s <.01), and in-group identification did not moderate these associations (*p*s >.27). The coping strategy self-blame mediated the association between active harm and stress. Substance use and self-blame mediated the association between passive harm and stress, whereas venting, behavioral disengagement, denial, self-blame, and social support mediated the association between everyday discrimination and stress. A direct effect remained for passive harm and everyday discrimination.

**Conclusion:**

The present study revealed that Russian immigrants encounter different forms of ethnic discrimination, and that this is associated with higher levels of stress. This association was partly explained by coping and social support, illustrating possibilities for interventions aimed at improving the use of adaptive coping strategies and promoting social support-seeking for Russian immigrants.

## Introduction

A growing body of research indicates that ethnic discrimination is associated with negative effects on both mental and physical health (1–3). Defined as unfair treatment that is attributed to a person’s ethnicity (4), ethnic discrimination poses threats to the well-being of most racial and ethnic minority groups (5). In addition to the direct association between ethnic discrimination and health, ethnic discrimination also leads to increased levels of stress, thus indirectly contributing to an impairment in mental and physical health (2, 6–8).

Research on the link between ethnic discrimination and stress has focused on potential protective factors, such as in-group identification (9, 10), which may alleviate stress when experiencing acts of ethnic discrimination. Furthermore, investigations have examined the use of individual coping strategies and perceived social support following the experience of ethnic discrimination (11, 12). Individual coping and perceived social support refer to cognitions and behaviors used to mitigate the stressful effects of perceived ethnic discrimination (13–15).

Since the 1970s, there have been indications of an overall reduction in overt ethnic discrimination and a simultaneous rise in covert, subtle, and benevolent forms from studies conducted in North America (16–18) and Europe (19, 20). Covert forms of ethnic discrimination have also been investigated under the label ethnic/racial microaggressions (18). Those were defined as verbal, behavioral, or environmental offenses against members of ethnic minority groups (21). This shift over to more covert forms has been assumed to be largely due to changing social norms and legislative interventions (22, 23). It is therefore important to account for the difference between distinct forms of ethnic discrimination. Most studies, however, have examined the effects of overt forms, while subtle and covert ethnic discrimination has not been investigated as frequently. The few studies to have investigated subtle and covert forms of discrimination also seem to indicate detrimental effects on individuals (24, 25). The behaviors from intergroup and affects and stereotypes-map [BIAS map; (26)] is a theoretical model that differentiates four forms of discrimination which are based on fundamental dimensions of social perception of group membership [for a recent review, see (27, 28)]. Two of these forms have negative consequences for the group member: active harm, which describes interpersonal acts with the intention to hurt or cause harm, and passive harm, which is a demeaning or diminishing behavior, and includes ignoring or neglecting. The two other forms are facilitatory behaviors and are assumed to lead to—ostensibly—favorable outcomes for the outgroup (26). The aim of active facilitation is to explicitly assist or interact with a group in a benevolent way; and passive facilitation describes behavior in which cooperation and association with a group is merely tolerated in the service of other goals [for a comprehensive review, see (27)]. Additionally, everyday discrimination, e.g., in restaurants, governmental institutions, or while applying for a job or a loan, is equally important to consider, as it continues to happen frequently (29–31). Everyday discrimination has restricting effects on the participation in several domains of daily life and, moreover, on fundamental basic necessities such as access to housing or job markets [e.g., (32)].

In-group identification was postulated to be an important factor that potentially moderates the consequences of ethnic discrimination (10). Within the social identity theory framework, in-group identification has also been referred to as ethnic identity [e.g., (33)]; we will use these terms synonymously. According to the social identity theory, perceived group membership is an important part of an individual’s self-concept (34), and high levels thereof can provide people with the resources to counteract the harm caused by discrimination. The rejection-identification model (35) depicts this buffering process as mediation and states that although perceived discrimination is negatively related to health, it may also enhance in-group identification, in turn having a positive effect on health (36). Most studies, however, investigated the moderating effect of in-group identification based on the model of McCoy and Major (37), in which the consequences of discrimination are determined by how strongly the individual identifies with the group. Indeed, in-group identification has been shown to buffer distress from ethnic discrimination (9, 38), but higher identification was also reported to increase stress and decrease well-being (39). It is therefore unclear whether high in-group identification protects the self-concept of the victim from the consequences of discriminatory acts [for a review, see (36)]. If membership of a certain group that suffers from discrimination is an important aspect of the self-concept, then acts of discrimination might also be more salient to oneself (40).

Individual coping refers to different cognitive or behavioral efforts that one uses to manage situations that are appraised to exceed, strain, or tax personal resources (41). According to the transactional model of stress and coping (41), and also the biopsychosocial model of racism as a stressor (11) and Harrell’s (12) racism-related stress model, individual coping acts a mediator between stressful events (such as instances of ethnic discrimination) and stress responses. How people cope with instances of ethnic discrimination may have either positive or negative impacts on mental and physical health. For instance, in the context of ethnic discrimination, substance use can be seen as a maladaptive coping mechanism, as it may buffer short-term levels of stress but contributes to the detrimental effects discrimination has on health [e.g., (42)]. Venting one’s anger after an experience of discrimination has been associated with higher levels of stress (14, 43) and may also act as a maladaptive coping strategy in this context. Similar maladaptive effects were found for behavioral disengagement (43) and acceptance (44). Furthermore, the tendency not to think about experienced incidents of discrimination, to deny them, or to avoid them (i.e., avoidance coping) was found to heighten their negative effects on distress and self-esteem (45–48) and on life satisfaction (49). In contrast, adaptive coping strategies, such as religious coping (50), were reported to reduce stress levels after experiences of discrimination. Problem-focused coping (i.e., personal and formal confrontation) was also associated with lower stress levels and fewer depressive symptoms [e.g., (51–54)].

The availability of interpersonal social support has been shown to be beneficial for well-being and psychological adjustment (55), particularly in immigrant populations (29, 44, 56–60). In the context of ethnic discrimination, social support might help people to cope with instances of ethnic discrimination, subsequently lowering distress (13, 36). Instances of ethnic discrimination are often discussed with family and friends after they have happened [e.g., (51, 61)], and a supportive social network may therefore be crucial in terms of adaptation to and reduction of the stress elicited by discrimination [for a review, see (13)]. However, the results are inconsistent, with one study reporting buffering effects of social support on the association between discrimination and psychological distress (62) and others only finding a buffering effect on depressive symptoms and not on distress (44, 58, 59). Overall, therefore, evidence for the buffering role of social support remains inconclusive [for a review, see (3)].

While some studies assessed how social support helps people to cope with stress in general (without focusing exclusively on ethnic discrimination), Clark (63) argued that social support must be measured in a stimulus-specific manner, i.e., by capturing coping that is specific to a certain stressor—in this case ethnic discrimination—in order to reveal a possible influence. This may explain the aforementioned discrepant findings regarding social support, as many studies did not include a stimulus-specific measurement of social support. A further important factor that has often been overlooked in previous research refers to how different forms of discrimination might have different effects on victims, and how this translates into efforts to cope with perceived ethnic discrimination.

### Present Study

We set out to examine the association between perceived ethnic discrimination—considering in-group identification and coping—in a sample of Russian immigrants in Germany. Russian immigrants have received very little attention in this context. For instance, in a meta-analysis of the literature examining the relationship between reported racism and mental and physical health outcomes (1), only three out of the 333 included studies focused exclusively on Russian immigrants. Despite this, Russian immigrants make up considerably sized immigrant groups in the United States, Israel, Finland, Greece, and Cyprus (64). In Germany, they constitute the third-largest group of immigrants, with 1.8 million members [i.e., first- or second-generation; (65)]. A majority of these individuals have a German family background, as their German ancestors have settled all across Europe since medieval times (66). Since 1953, and markedly so following the dissolution of the USSR, ethnic Germans and their descendants have been permitted to repatriate to Germany and have been able to receive benefits such as financial support and automatic citizenship. However, Russian immigrants living in Germany have reported similar encounters of ethnic discrimination to other ethnic groups (67–69). Compared to Germans without migration background, Russian immigrants reported poorer health status (70), higher prevalence rates of cardiovascular diseases (71) or risk factors for cardiovascular diseases (72, 73), and higher rates of mental health problems (66, 74).

**Figure 1:**
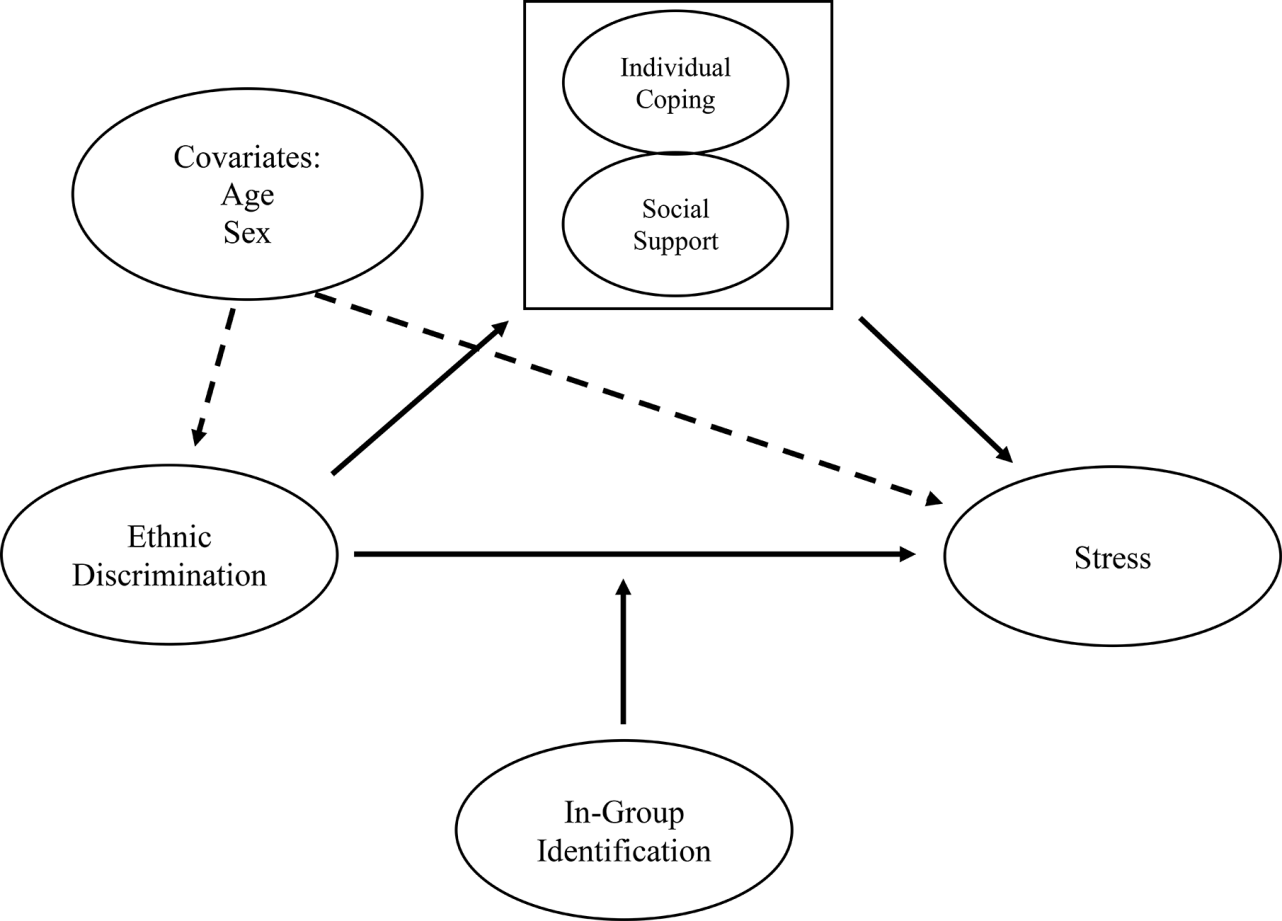
Mediation and moderation models for predicting levels of stress by forms of ethnic discrimination *via* individual coping and social support (mediators), with the strength of in-group identification as a moderator.

To our knowledge, no study to date has investigated the associations between different forms of ethnic discrimination and stress, as well as moderating and mediating factors in Russian immigrants. As such, it is unclear whether in-group identification buffers or heightens the perceived stress caused by instances of ethnic discrimination in this population. In the present study, we chose to investigate overt and covert discrimination by using two of the four behavioral tendencies of the BIAS map (i.e., active harm and passive harm), as in contrast to the facilitatory dimensions, these have negative consequences for individuals. Furthermore, we assessed ethnic discrimination in everyday settings. Everyday discrimination encompasses elements of exclusionary behaviors in daily life that may be perceived indirectly, without personal contact, intent, or demeaning nature (75), constituting a form of discrimination that may be distinct from active and passive harm. We chose to investigate this form of ethnic discrimination separately as it—reportedly—continues to happen frequently [e.g., (76)] and was previously found to be a distinct form of ethnic discrimination in a sample of Turkish immigrants in Germany (10). The study thus investigated the potential moderating influence of in-group identification on the relationship between three different forms of ethnic discrimination (active harm, passive harm, and everyday discrimination) and stress. Additionally, we investigated whether discrimination-specific coping and social support mediated the relationship between the different forms of ethnic discrimination and perceived stress (see **Figure 1** for an overview).

Our research hypotheses were as follows:

1. The relationship between different forms of ethnic discrimination and perceived stress is moderated by in-group identification.
2. The positive relationship between different forms of ethnic discrimination and perceived stress is mediated by discrimination-specific individual coping and social support. We assume that emotion-focused coping (e.g., venting and substance use) and avoidance coping (e.g., behavioral disengagement and denial) will be maladaptive (i.e., associated with higher perceived stress), whereas problem-focused coping, religious coping, and social support will be adaptive (i.e., associated with lower perceived stress).

## Methods

### Procedure

The questionnaires were administered online at two time points, between May and August 2012 and between January and May 2015. The participants of both sampling time points were independent from each other, i.e., the data is of cross-sectional nature. For the purpose of the current study, we merged the datasets from 2012 (*N* = 159) and 2015 (*N* = 149). The study was approved by the Committee for Ethics at the Department of Psychology, Philipps University Marburg, Germany. Informed consent was obtained from all participants prior to their participation.

Participants were recruited *via* advertisements in supermarkets and institutions (e.g., authorities of local Russian-Orthodox and Jewish communities) in the Federal states of Hesse and North Rhine-Westphalia in Germany. Further recruitment occurred in social and professional networks, online communities, and mailing lists which span across Germany. To portray the population in detail as well as gain generalizable results, we included both first and second-generation immigrants who, in 2018, made up 79 and 21% of Russian immigrants living in Germany, respectively (65). The recruitment strategy did not differ between the two time points.

### Participants

Our sample comprised 308 Russian immigrants (204 females; age *M* = 31.8, *SD* = 10.6, 18–77 years) who had been living in Germany for an average of 15.5 years (*SD* = 7.3); 9% (*n* = 29) were second-generation immigrants, i.e., born in Germany. Twenty-four percent of the participants had a university degree, and 15% percent did not possess any school-leaving qualifications. Fifty-nine percent were employed, while the remainder were enrolled in university or currently unemployed. Eighty-five percent were in a relationship or married. The participants of the second generation were younger (first generation: *M* = 32.8 years, second generation: 23.0 years, *p* <.001) and higher educated (university degree: first generation, 21%; second generation, 52%, *p* <.001) than the participants of the first generation.

Compared to the subsample from 2012, the subsample from 2015 was younger (*M* = 27.5 versus *M* = 35.9 years, respectively, *p* <.001), higher educated (46% versus 4% had a university degree, *p* <.001), and fewer people were employed (50% versus 68%, *p* = .002). Furthermore, fewer people were in a relationship or married in 2015 (42%) than in 2012 (73%, *p* <.001).

### Measures

All questionnaires were translated into Russian using the forward-backward method by native speakers living in Germany, with the exception of the Perceived Stress Scale, which already existed in Russian (77). Consequently, all questionnaires were offered in Russian and in German.

First, participants provided information on their age, sex, education, and relationship status.

Two different questionnaires were used to measure discrimination. The BIAS-treatment scale [BIAS-TS; (78)] is a measure assessing different forms of discrimination. It is based on the BIAS map (26) and detects four different kinds of harmful and facilitatory behavior that individuals may encounter: active harm (intention to hurt), passive harm (ignorance, undermining the social value), active facilitation (benefiting a group), and passive facilitation (instrumental collaboration to pursue one’s own aims). We applied the BIAS-TS-Short Form with three items for each scale, and items were rated on a 7-point scale (1 = have never experienced this, 7 = often experience this). As the BIAS-TS had never been used in a sample of Russian immigrants, confirmatory factor analysis (CFA) was conducted using the lavaan package (79) for R version 3.6.0. The four factors (active harm, passive harm, active facilitation, and passive facilitation) were confirmed, with acceptable fit [χ^2^(48) = 133.34, *p* <.001; RMSEA = .076, CFI = .914, SRMR = .068]. This model with four latent factors fitted the data significantly better than a model with only a single latent factor [Δχ^2^(6) = 388.38, *p* <.001] or a two-factor active and passive model [Δχ^2^(5) = 263.42, *p* <.001]. Cronbach’s alpha was .75 for active harm and .68 for passive harm (active and passive facilitation are not used in the current study, as facilitatory behaviors do not fit our definition of discrimination).

Everyday experiences of discrimination were measured by a combination of seven items from three different studies (10, 68, 80). The items were based on the Everyday Discrimination Scale (54) and measured discrimination in an everyday context. Specifically, we used the items assessing insults, not getting hired or promoted, receiving poorer service at restaurants/stores, and not being able to get apartments/houses for rent, which were shown to be meaningful for Russian immigrants in a study by Salentin (68). Furthermore, we included two items assessing inadequate care from a doctor and being treated worse than others in government institutions/agencies, as everyday discrimination was reported to happen frequently in these contexts by minority groups in Germany (81). Items were answered on 7-point scales (1 = have never experienced this, 7 = often experience this). Principal axis factor analysis revealed one factor with an eigenvalue of 3.34 and all items had factor loadings above .39 on this factor, explaining 47.64% of the variance. Cronbach’s alpha for everyday discrimination was .81.

We conducted a principal axis factor analysis (with direct oblimin rotation) to ensure that the three forms of ethnic discrimination (i.e., active harm, passive harm, and everyday discrimination) were distinct factors. Sphericity (χ^2^(78) = 1259.92, *p* <.001) and size of the KMO (.80) were acceptable. Factor analysis revealed three factors with eigenvalues > 1, explaining 57% of the variance. The three factors consisted of 1) the three active harm items (loadings >.52), 2) the three passive harm items (loadings >.53), and 3) the seven everyday discrimination items (loadings >.49), respectively. No cross-loadings above .25 were found. These results thus confirmed the factorial distinctiveness of our three scales measuring ethnic discrimination.

The Perceived stress scale (PSS) by Cohen, Kamarck, and Mermelstein (82) measures perceived stress in the previous month. We used a short version of the scale with ten items (83). All items were rated on 5-point scales (1 = never, 5 = always). In the original version, participants are asked to report how often they felt a certain way (e.g., “In the last month, how often have you felt that you were unable to control the important things in your life?”). We extended the period to the last year in order to assess a period that is congruent with the BIAS-TS. Cronbach’s alpha of the PSS lay at .88 in our study.

Participants completed four items measuring their identification with the group of Russians. These items were adapted from two studies (84, 85) and have been used previously in a Russian population (84). A typical item was: “To be a Russian is an important aspect of my person”, with responses ranging from 1 (don’t agree at all) to 7 (agree completely). Principal axis factor analysis revealed one factor with an eigenvalue of 3.06 and all items had factor loadings above .78 on this factor, explaining 76.42% of the variance. Cronbach’s alpha was .90 for in-group identification.

To assess individual coping, we used the Brief COPE (86), which is the short form of the original COPE (87) and is based on the assumptions of the transactional model of stress (41). The Brief COPE contains 28 items and 14 scales. In the present study, we used the items of twelve scales (24 items); the scales Emotional Support and Instrumental Support were omitted as one can assume that the availability of social support—as measured with the ENRICHD Social Support Inventory in our study (see below)—can predict the seeking of social support (88–90). All items are rated on a 5-point scale (1 = never, 5 = always). We formulated the instructions to measure coping specifically after perceived experiences of discrimination, i.e., discrimination-specific individual coping [c.f., (8, 91)].

In order to generate scales, a principal axis factor analysis with direct oblimin rotation was conducted. Bartlett’s test of sphericity [χ^2^(276) = 3348.00, *p* <.001] and size of the KMO (.79) were acceptable. Factor analysis revealed eight factors with eigenvalues of >1, explaining 72% of the variance. Due to low communalities or double factor loadings, items 7, 17, 19, and 24 were excluded from further analyses. The factor active coping found by Knoll, Rieckmann, and Schwarzer (92) was replicated (see factor 1 in **Table 1**). The other factors were named as follows: substance use (factor 2), venting (factor 3), humor and positive reframing (factor 4), behavioral disengagement (factor 5), religion (factor 6), denial (factor 7), and self-blame (factor 8). The pattern matrix and Cronbach’s alpha of each subscale is depicted in **Table 1**.

**Table 1:** Pattern matrix of the scales of the Brief COPE and Cronbach’s alpha.

Item	Active coping	Substance use	Venting	Humor and positive reframing	Behavioral disengagement	Religion	Denial	Self-blame
(27) I’ve been thinking hard about what steps to take.	**.74**							
(14) I’ve been trying to come up with a strategy about what to do.	**.72**							
(7) I’ve been taking action to try to make the situation better.	.59				-40			
(2) I’ve been concentrating my efforts on doing something about the situation I’m in.	**.58**							
(1) I’ve been turning to work or other activities to take my mind off things.	**.54**							
(21) I’ve been doing something to think about it less, such as going to movies, watching TV, reading, daydreaming, sleeping, or shopping.	.37				.31			
(11) I’ve been using alcohol or other drugs to help me get through it.		**.98**						
(4) I’ve been using alcohol or other drugs to make myself feel better.		**.86**						
(23) I’ve been expressing my negative feelings.			**.84**					
(9) I’ve been saying things to let my unpleasant feelings escape.			**.60**					
(18) I’ve been making jokes about it				**.93**				
(30) I’ve been making fun of the situation.				**.55**				
(17) I’ve been looking for something good in what is happening.	.31			.37				
(22) I’ve been accepting the reality of the fact that it has happened.				**.33**				
(12) I’ve been trying to see it in a different light, to make it seem more positive.				**.32**				
(6) I’ve been giving up trying to deal with it.					**.66**			
(16) I’ve been giving up the attempt to cope.					**.56**			
(24) I’ve been trying to find comfort in my religion or spiritual beliefs.						**1.00**		
(29) I’ve been praying or meditating.						**.69**		
(8) I’ve been refusing to believe that it has happened.							**.80**	
(3) I’ve been saying to myself “this isn’t real.”.							**.63**	
(28) I’ve been blaming myself for things that happened.								**.80**
(13) I’ve been criticizing myself.								**.78**
(26) I’ve been learning to live with it.								
**Cronbach’s alpha**	.79	.92	.79	.65	.65	.81	.67	.90

Principal axis factor analysis with oblimin rotation. Factor loadings <.30 are not indicated. Factor loadings in bold indicate that the item was used for the computation of the respective factor. Cronbach’s alpha is calculated without items 7, 17, 19, and 24, which were excluded from further analysis.

We used the ENRICHD social support inventory [ESSI; (93)] to measure social support. In contrast to the six-item original version of the questionnaire, the German version includes only five items, without the item assessing instrumental support (94). As instrumental social support is important in the context of discrimination [e.g., (95)], we added an item measuring this aspect (“Is there someone available to you who can provide practical and concrete help with problems (e.g., take you to the doctor?”) from the Swiss Household Panel (96). For all items, the instructions were specifically formulated to refer to discrimination, similar to the instructions of the Brief COPE. Participants rated the availability of social support on a 5-point scale (1 = never, 5 = always). Principal axis factor analysis revealed one factor with an eigenvalue of 3.83, and all items had factor loadings above .64, explaining 63.94% of the variance. Cronbach’s alpha was .91.

### Data Analysis

Analyses were conducted using IBM SPSS 24. Moderation and mediation models were tested using the PROCESS plugin (version 2.16.3) for SPSS 24 (97). Three regression analyses were conducted, with the three forms of discrimination, in-group identification, and the respective discrimination x identification interaction term. Predictors and potential moderators were mean-centered (98). We tested three parallel multiple mediation models in which individual coping and social support were entered as mediators of the relationship between the three forms of discrimination (active harm, passive harm, and everyday discrimination) and perceived stress. Bias-corrected bootstrapping (99) was used to test the mediating effect of our proposed variables on the relations between the forms of discrimination and perceived stress. A series of steps were undertaken to test our models. First, the forms of discrimination were regressed onto our proposed mediators. Second, our mediators were entered into a regression predicting perceived stress, controlling for the predictor (i.e., the form of discrimination of each model). Third, the indirect effects of all the individual mediators were computed as a full model. We then retained relevant mediators (i.e., with a bootstrapped CI not including zero) and dropped the remaining non-relevant mediators for each model. A final model was subsequently computed including only relevant mediation paths. Total and direct effects were reported based on final models. For all analyses, a 95% confidence interval (CI) with 10,000 bootstrapping samples was used. The proportion of explained variance of the outcome in our final models (including the predictors and mediators simultaneously) is depicted with *R* ^2^ statistics. Following general convention (100), an *R* ^2^ of 0.02 was considered as a small, 0.13 a moderate, and 0.26 a large proportion of explained variance. Age and sex were included as covariates in all moderation and mediation models.

## Results

All bivariate correlations of the variables included in our models are depicted in **Table 2**. The age of participants showed a significant positive association with all three forms of perceived ethnic discrimination (*r*s = .13–.28), but not with perceived stress (*r* = -.08). Correlations between sex (coded as 1 = male, 2 = female) and active harm (*r* = -.18) were significant. Male participants reported a higher frequency of active harm. In-group identification was positively—and significantly—associated with perceived stress (*r* = .15).

**Table 2:** Bivariate correlations between model variables.

	(1)	(2)	(3)	(4)	(5)	(6)	(7)	(8)	(9)	(10)	(11)	(12)	(13)	(14)	(15)	(16)
**(1) Everyday discrimination**	–															
**(2) Active harm**	.36***	–														
**(3) Passive harm**	.28***	.25***	–													
**(4) Perceived stress**	.18**	.08	.22***	–												
**(5) In-group identification**	.19***	.06	.19***	.15**	–											
**(6) Active coping**	.33***	.10	.28***	.20***	.21***	–										
**(7) Substance use**	.10	.10	.23***	.31***	.003	.18**	–									
**(8) Venting**	.18**	.09	.11	.22***	.08	.40***	.26***	–								
**(9) Humor**	.15**	.05	.12*	.10	.19**	.42***	.07	.27***	–							
**(10) Behavioral disengagement**	.16**	.03	.08	.27***	.17**	.24***	.14*	.19***	.31***	–						
**(11) Religion**	.17**	.22***	.17**	.06	.07	.18**	.23***	.10	.12*	.13*	–					
**(12) Denial**	.19**	.09	.15**	.05	.03	.39***	.22***	.36***	.26***	.23***	.21***	–				
**(13) Self-blame**	.20***	.10	.27***	.31***	.07	.47***	.25***	.25***	.24***	.36***	.30***	.29***	–			
**(14) Social support**	-.23***	-.07	-.12*	-.18**	.01	-.24***	.01	-.03	-.04	-.16**	.05	-.04	-.13*	–		
**(15) Age**	.27***	.13*	.15**	-.08	-.10	.14*	-.05	-.002	.06	.001	-.04	.09	.04	-.42***	–	
**(16) Sex (1 = male, 2 = female)**	-.09	-.18*	-.07	.14	.12	-.01	.03	.11	-.003	.14*	.03	.06	.06	.22***	-.14*	–
***M***	1.99	1.35	2.51	2.92	4.30	2.78	1.26	2.54	2.51	1.90	1.65	2.01	1.99	3.82	31.81	1.66
***SD***	1.09	0.71	1.30	0.68	1.86	0.84	0.61	0.93	0.71	0.81	0.87	0.89	0.96	0.94	10.64	0.47

*p <.05, **p <.01, ***p <.001.

The participants from the first and second-generation did not differ regarding their scores of active harm, passive harm, or perceived stress (*p*s >.191). First-generation immigrants, however, reported a higher frequency of everyday discrimination (*M* = 2.04) than second-generation immigrants (*M* = 1.52, *p* = .001) and a higher score of in-group identification (*M* = 4.37 versus *M* = 3.59, *p* = .017). The subsample from 2015 had lower scores in all measurements of the three forms of ethnic discrimination (active harm: *M* = 1.22 versus *M* = 2.47; passive harm *M* = 2.32 versus *M* = 2.69; everyday discrimination: *M* = 1.77 versus *M* = 2.20, all *p*s <.013) than the sample assessed in 2012. Furthermore, the subsample from 2012 reported higher in-group identification (*M* = 4.51) than the subsample from 2015 (*M* = 4.08, *p* = .040). The two samples, however, did not differ regarding perceived stress (*p* = .847).

### Moderation of the Relationship Between Discrimination and Stress with In-Group Identification as Moderator Variable

None of the three interaction terms were significant (active harm: *b* = 0.03, *p* = .22; passive harm *b* = 0.009, *p* = .58; everyday discrimination: *b* = -0.02, *p* = .27), indicating that in-group identification did not moderate the associations between the three forms of discrimination and perceived stress. To investigate whether the potential moderation may be influenced by the different generational statuses and sampling time points of our sample, we analyzed the models separately for first- and second-generation participants and the subsamples from 2012 and 2015. Again, in-group identification did not moderate the associations between any forms of discrimination and perceived stress (first generation: *p*s >.23, second generation: *p*s >.52, subsample from 2012: *p*s >.18, subsample from 2015: *p*s >.14). Consequently, in-group identification was not considered as a control variable in the further analyses.

### Mediation of the Relationship Between Discrimination and Stress by Coping and Social Support

We tested three parallel multiple mediation models, one for each form of discrimination (active harm, passive harm, and everyday discrimination) with the eight different coping strategies and social support as mediators. See **Supplementary Table 1** for unstandardized coefficients and indirect effects, and **Table 3** for the total and direct effects of all three models.

**Table 3:** Results from mediation analysis for the total and direct effects of all models.

Model	Total effect	*SE*	Direct effect	*SE*	*F* (*df*)	*R* ^2^
Model 1: Active harm *via* coping -> stress	0.11	0.06	0.08	0.05	10.75*** (4, 303)	.12
Model 2: Passive harm *via* Coping -> stress	0.13***	0.03	0.07*	0.02	13.85*** (5, 302)	.19
Model 3: Everyday discrimination *via* coping -> stress	0.14***	0.04	0.08*	0.04	10.56*** (8, 299)	.22

*p < .05, ***p < .001.

**Figure 2:**
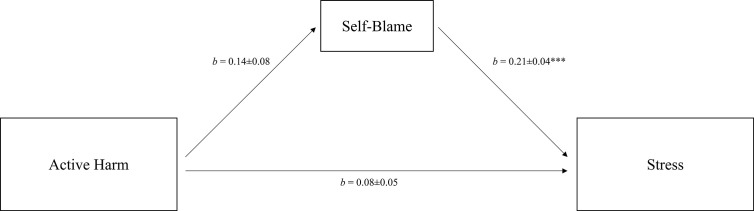
Mediation of the relation between active harm and perceived stress *via* individual coping. Total effect: *b* = 0.11, *SE* = 0.06, *p* = .051. Control variables: age and sex. *R* ^2^ = 0.12. *b* = unstandardized coefficients ± *SE*. ***p < .001.

For active harm, we only found an indirect effect on perceived stress through self-blame (see **Figure 2** for the final model). The total effect of active harm on stress was not significant (*b* = 0.11, *p* = .051), and the inclusion of self-blame as a mediator reduced it to a direct effect of *b* = 0.08 (*p* = .14). The final model explained a small proportion of the variance in perceived stress (*R* ^2^ = .12).

**Figure 3:**
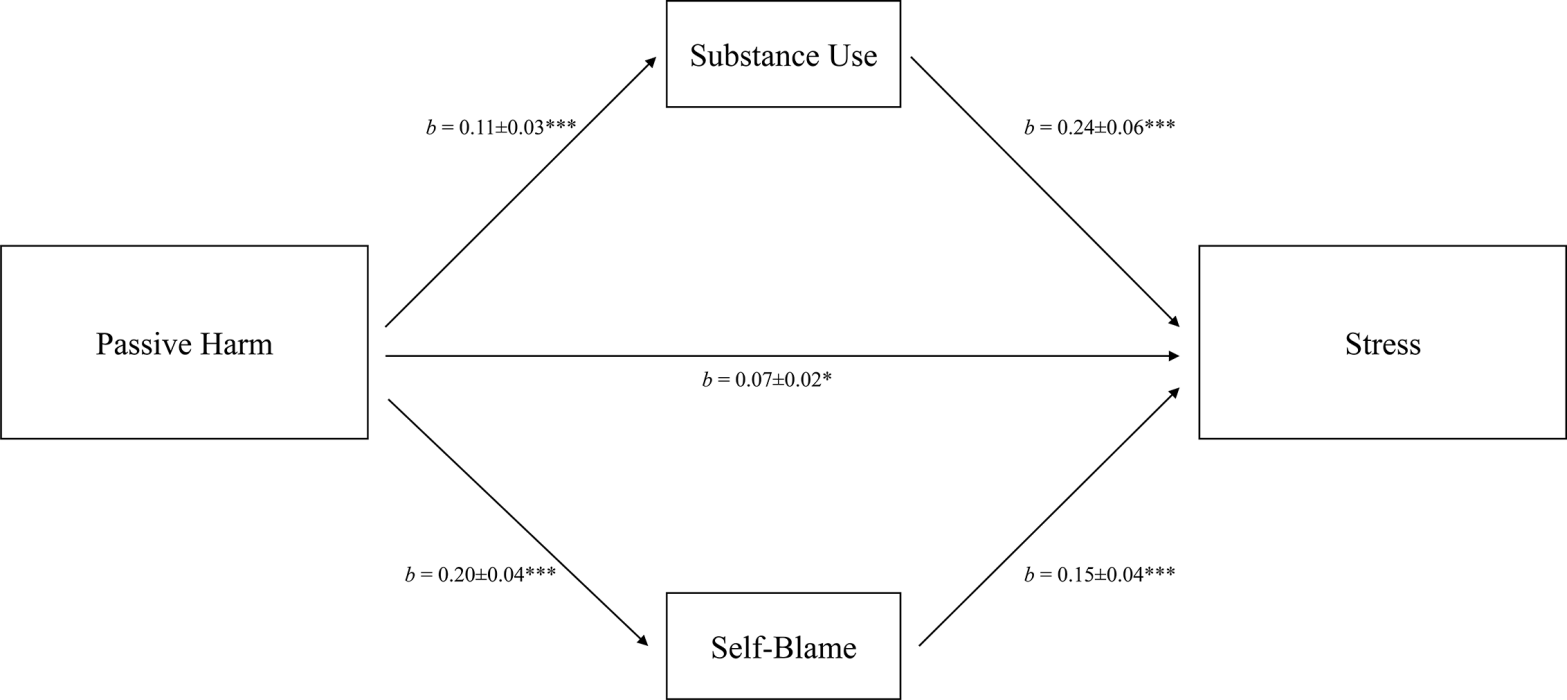
Mediation of the relation between passive harm and perceived stress *via* individual coping. Total effect: *b* = 0.13, *SE* = 0.03, *p* <.001. Control variables: age and sex. *R* ^2^ = 0.19. *b* = unstandardized coefficients ± *SE*. ***p <.001.

Passive harm showed an indirect effect on perceived stress through substance use and self-blame (see **Figure 3**), which reduced the total effect of passive harm from *b* = 0.13 (*p* <.001) to a direct effect of *b* = 0.07 (*p* = .020). The variance explained by the final model (*R* ^2^ = .19) indicated a moderate effect size.

**Figure 4:**
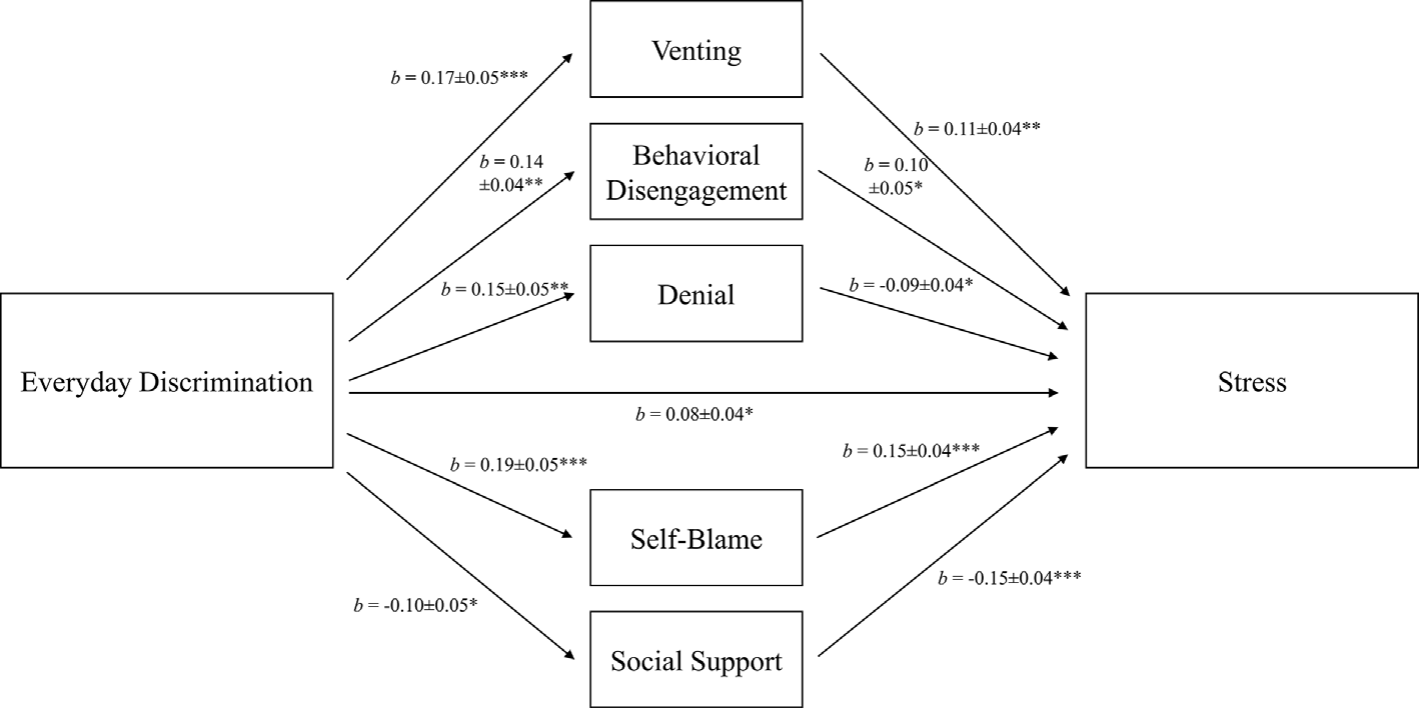
Mediation of the relation between everyday discrimination and perceived stress *via* individual coping. Total effect: *b* = 0.14, *SE* = 0.04, *p* <.001. Control variables: age and sex. *R* ^2^ = 0.22. *b* = unstandardized coefficients ± *SE*. *p <.05, **p <.01, ***p <.001.

We found significant indirect effects of everyday discrimination on perceived stress through venting, behavioral disengagement, denial, self-blame, and social support (see **Figure 4** for the final model). The final model indicated that the inclusion of mediators reduced the total effect of everyday discrimination, with *b* = 0.14 (*p* <.001), to a direct effect of *b* = 0.08 (*p* = .027). The variance explained by the final model (*R* ^2^ = .22) indicated a moderate effect size.

## Discussion

Experiences of discrimination are associated with increased stress and reduced well-being. The present study aimed to investigate the associations between three different forms of ethnic discrimination (active harm, passive harm, and everyday discrimination) and perceived stress in a sample of Russian immigrants living in Germany. We explored the stress-buffering effect of in-group identification and the use of various discrimination-specific coping strategies (active coping, substance use, venting, humor and positive reframing, behavioral disengagement, religion, denial, and self-blame) as well as perceived social support.

Consistent with previous research (1, 2), the findings of the present study revealed that greater exposure to ethnic discrimination was related to higher levels of stress. Passive harm, a subtle and paternalistic form of ethnic discrimination, was reported most frequently in our sample of Russian immigrants, followed by everyday discrimination and active harm. This frequency distribution is similar to other studies which reported that the frequency of more subtle forms of discrimination has increased over time, whereas blatant forms have become less frequent [e.g., (16, 101)]. We found that everyday discrimination was prevalent in our sample; this fits the notion that discrimination remains a pervasive factor in housing, education, employment, and health care (30, 102–104).

Active harm, which is characterized by a perception of blatant, overt ethnic discrimination, was not associated with perceived stress in the present study. Ong, Fuller-Rowell, and Burrow (105) found a positive association between the frequency of perceived discrimination and induced stress. Thus, the low frequency of blatant acts of discrimination found in the investigated sample may explain the lack of relationship with stress. Moreover, the prevalence of reported active harm in our subsample from 2015 was much lower than in the subsample from 2012. One could argue that this difference has influenced our results, as possible associations between active harm and stress in the subsample from 2012 could have been averaged out by merging the data with the subsample from 2015. However, separate correlation analyses (results not reported) for the subsamples from 2012 and 2015 showed no associations between active harm and stress. We can, therefore, preclude the possibility that this temporal variation affected the overall association between active harm and stress. Instances of passive and subtle ethnic discrimination—rather than overt forms—may be a preeminent factor leading to stress and in turn to stress-related disorders, as such instances may be ubiquitous, very easily dismissed by perpetrators, and more frequent.

In-group identification did not moderate the relationship between any form of discrimination and the level of stress in our sample. This is contrary to some studies reporting a relationship between in-group identification or ethnic identity and the perception of discrimination (106–110), which may in turn suggest a moderating effect of in-group identification on the relationship between discrimination and level of stress. Nonetheless—and in line with our results—one review found no buffering effect of in-group identification in 10 of 12 studies investigating coping with racism (13). Even though the ethnic identity of Russian immigrants participating in our study was relevant to them (as indicated by the high mean score on the in-group identification scale), it neither strengthened nor weakened the association between ethnic discrimination and perceived stress. Furthermore, a possible mediation of the association between ethnic discrimination and stress *via* in-group identification, as assumed by the rejection-identification model (35), can be precluded as an alternative explanation for our results. Contrary to the predictions of this model, in-group identification did not reduce stress, but was rather positively associated with stress in our sample of Russian immigrants. In-group identification is considered to collectively develop over time, through participation in social and cultural practices of one’s group, and through social comparisons with both the in-group and the out-group (9). The history of Russian immigration to Germany is in part a relatively recent phenomenon (i.e., having occurred since the dissolution of the USSR). Furthermore, Russian immigrants in Germany are characterized by a heterogeneity of immigration backgrounds (i.e., differences in religious faith, mixed ethnic heritage, and country of origin). A mixed historical and experiential knowledge about the in-group could therefore explain the lack of a stress-buffering effect in the present study (111).

As described in the theoretically grounded models of coping as a mediator between experiences of ethnic discrimination and stress (11, 12), mediating effects of coping and/or social support were found between all three investigated forms of discrimination and perceived stress. In response to passive harm, Russian immigrants tended to use substances such as alcohol and engaged in self-blame. Similarly, self-blame was found after experiences of active harm. Following ethnic discrimination in everyday situations, multiple coping strategies were used (i.e., behavioral disengagement, self-blame, venting, denying, and social support). Social support was negatively associated with everyday discrimination, i.e., it was used to a lesser extent after perceiving this form of ethnic discrimination. The number of relevant coping strategies in the link between everyday discrimination and stress was higher than for active harm (one strategy) and passive harm (two strategies). An explanation may be that the items assessing everyday discrimination were more concrete and more salient for our participants—when compared to the more general behaviors in the BIAS-TS. Moreover, we aimed to assess a wide range of discriminatory experiences with close proximity to everyday life. It may be due to the high salience and the proximity to everyday life—and maybe also to other stressful experiences in daily life—that a broader range of coping strategies was associated with the stress level in these situations. Possibly, everyday discrimination is less specific with regard to applied coping strategies than other forms of ethnic discrimination. The number of significant coping strategies in our assessment of active and passive harm was, however, similar to other studies that used the Brief COPE (43, 91). The effects of discrimination on stress in our sample occurred largely (passive harm, everyday discrimination) or entirely (active harm) through indirect effects, and these associations remained after holding age and sex constant. However, passive harm and everyday discrimination also showed direct associations with perceived stress beyond the influence of coping strategies.

Of the nine coping strategies investigated in this study, six were relevant mediators of the relationship between different forms of discrimination and stress. As hypothesized, emotion-focused strategies (i.e., venting, substance use, self-blame), as well as one avoidant strategy (behavioral disengagement), were maladaptive. Laypersons often assume venting to be a productive strategy to handle negative emotions (112). However, other studies also found that higher levels of venting after discriminatory experiences led to higher psychological distress (43, 91).Venting one’s anger does not seem to help regulating negative emotions after ethnic discrimination, but rather seems to result in a prolongation of those negative emotions (113). Our findings also suggest that behavioral disengagement after discriminatory events did not lead to effective stress management, which is consistent with other findings in this context (43, 114). It is assumed that behavioral disengagement was an indicator of learned helplessness (115), which could mean that victims of discrimination “gave up” or withdrew from actively dealing with stressors.

Only two strategies were found to buffer against stress following experiences of ethnic discrimination: perceived social support and denial, and these strategies only exerted an effect in response to perceived everyday discrimination. Contrary our expectations, we did not find a mediation by problem-focused and religious coping. Furthermore, everyday discrimination was associated with lower social support, even though this form of coping buffered perceived stress. Prelow, Mosher, and Bowman (116) reported that the perceptions of social support decreased in African American college students affected by racial discrimination and that this reduction in social support partially accounted for the detrimental effects of discrimination on psychological adjustment. One study of Russian immigrants in Finland found that the availability of social support networks was related to better psychological well-being (117). Nevertheless, other studies found either no mediation [in African Americans, (91); in native Hawaiians, (43)] or even a worsening effect of social support on distress [in Filipino Americans, (45)]. The mediating role of denial and its negative association with stress in our sample was striking, as it is commonly assumed that this avoidant coping strategy is psychologically taxing and debilitating, requiring permanent effort to deny the experiences of ethnic discrimination (45, 118, 119). One study found that African Americans who constantly denied unfair treatment against them had elevated resting blood pressure (61), and denial was associated with increased stress in samples of Latinos (118, 120). However, positive aspects of avoidance coping have also been acknowledged: Avoidance coping may protect against the perception and the processing of distressing information (121), and, in its milder form, denial may overlap with positive thinking (122). Furthermore, avoidance coping was found to be used more frequently in racially stressful than nonracially stressful events (123), possibly explaining the stress-buffering effect found in our study.

### Limitations

Our findings have to be considered in the light of potential limitations. Highly educated persons were overrepresented in our convenience sample (24% had a university degree compared to 11% in the population) and the mean age (32 years) was lower than the average age of Russian immigrants in Germany [41 years (65)], which may reduce the generalizability of our results. We recruited participants at two time points (i.e., 2012 and 2015) in order to extend our sample size and to attain results that are generalizable over time. This may be noted as a limitation, since the subsamples differed in sociodemographic variables. Furthermore, even though we could assume an indirect association between discrimination and levels of stress *via* coping and social support based on the theoretical background (11, 12, 41), this study was based on correlational data and did not allow for the examination of causal relationships. As mediation analysis does not permit claims regarding the direction of effects, it might be possible that the perceived stress of an individual was associated with the use of coping strategies and that this in turn affected the recall of discrimination experiences. Whereas the original PSS-10 assesses perceived stress during the last month (83), we aimed for concordance with the sampling frame of the BIAS-TS (1 year) and accordingly adapted the time frame of the PSS-10. While longer periods may be susceptible to recall bias in the appraisal of stressful events (124), the same limitation may account for the assessment of ethnic discrimination. In adapting the time frame of the PSS to the time frame of our main predictor measure (the BIAS-TS), we aimed to obtain the same conditions for these assessments and thus to avoid recall biases that only influence one variable and may unequally confound the reliability of the association. We decided against adapting the time frame of the BIAS-TS to only one month, as this may be a too short time frame to adequately assess ethnic discrimination. Moreover, research shows that even ethnic discrimination experienced a longer time back may have prolonged negative effects on health (125). Longitudinal studies investigating individual coping with discrimination on a long-term basis are needed to address these issues.

At the first page of the online assessment, participants could choose to fill in the questionnaires in either German or Russian, and the informed consent and all questionnaire where then presented in the chosen language. Very unfortunately and due to technical reasons, we could not determine how many participants chose which language. Thus, we were unable to test for measurement invariance or comparability of the two language versions. However, ninety percent of our sample rated their German language skills as “very good” or “good”, and the internal reliability (Cronbach’s alpha) of our core questionnaires (the three forms of discrimination, perceived stress, and in-group identification) did not differ between the skill categories for German language. The categories and respective internal reliability scores were as follows: very good (*n* = 205): α = .63–89; good (*n* = 69): α = .65–.90; and moderate or not very proficient (*n* = 32): α = .74–.92. This data may partially refute this limitation, as we assume that most participants in the current study chose the German version of our instruments due to their high proficiency in the German language and the internal reliabilities of the instruments were comparable between different German language skills. Nevertheless, we cannot fully rule out limitations due to measurement variance, as we did not test it.

## Conclusion

In sum, our findings add to the literature regarding negative effects of perceived ethnic discrimination on affected populations. The results suggest that different forms of perceived ethnic discrimination are indeed associated with stress. It should be recognized that Russian immigrants, an ethnic group that has been investigated relatively rarely, suffer from the impact of perceived ethnic discrimination, as we found evidence for the detrimental effects of subtle and everyday discrimination on the stress levels of Russian immigrants in Germany. The notion that different forms of ethnic discrimination may be experienced differently has not yet been extensively investigated. While the decline of blatant forms of ethnic discrimination may lead to the conclusion that discrimination of immigrants is of diminishing relevance, the present study underlines the harmful effects of more subtle forms of discrimination. Our study provides evidence for the possible effects of several maladaptive (substance use, venting, behavioral disengagement, self-blame) and adaptive (social support, denial) coping strategies following different forms of discrimination. Consequently, practitioners working with Russian immigrants affected by ethnic discrimination should encourage the seeking of social support and aim to reduce maladaptive coping strategies. Finally, we seek to raise institutional as well as public awareness of the consequences of discrimination against minority groups.

## Data Availability Statement

The raw data supporting the conclusions of this article will be made available by the authors, without undue reservation.

## Ethics Statement

The studies involving human participants were reviewed and approved by Committee for Ethics at the Department of Psychology, Philipps University Marburg, Germany. The patients/participants provided their written informed consent to participate in this study.

## Author Contributions

AG analyzed and interpreted the data and wrote the first draft of the manuscript. FA contributed to the design of the study, the interpretation of the data, and critically revised earlier versions of the manuscript. UN contributed to the interpretation of the data and critically revised earlier versions of the manuscript. RM was the principal investigator of the study, contributed to the design of the study, supervised data collection, helped with analyses and interpretation of the data, and was a major contributor in writing the manuscript. All authors contributed to the article and approved the submitted version.

## Conflict of Interest

The authors declare that the research was conducted in the absence of any commercial or financial relationships that could be construed as a potential conflict of interest.

## References

[1] Paradies Y Ben J Denson N Elias A Priest N Pieterse A Racism as a Determinant of Health: A Systematic Review and Meta-Analysis. PloS One (2015) 10(9):e0138511. 10.1371/journal.pone.0138511 26398658 PMC4580597

[2] Pascoe EA Smart Richman L Perceived discrimination and health: a meta-analytic review. Psychol Bull (2009) 135(4):531–54. 10.1037/a0016059 PMC2747726 19586161

[3] Schmitt MT Branscombe NR Postmes T Garcia A The consequences of perceived discrimination for psychological well-being: a meta-analytic review. Psychol Bull (2014) 140(4):921–48. 10.1037/a0035754 24547896

[4] Contrada RJ Ashmore RD Gary ML Coups E Egeth JD Sewell A Ethnicity-Related Sources of Stress and Their Effects on Well-Being. Curr Dir Psychol Sci (2000) 9(4):136–9. 10.1111/1467-8721.00078

[5] Brondolo E Beatty DL Cubbin C Pencille M Saegert S Wellington R Sociodemographic Variations in Self-Reported Racism in a Community Sample of Blacks and Latino(a)s. J Appl Soc Psychol (2009) 39(2):407–29. 10.1111/j.1559-1816.2008.00444.x

[6] Brown TN Williams DR Jackson JS Neighbors HW Torres M Sellers SL “Being black and feeling blue”: the mental health consequences of racial discrimination. Race Soc (2000) 2(2):117–31. 10.1016/s1090-9524(00)00010-3

[7] Mirsky J Slonim-Nevo V Rubinstein L Psychological Wellness and Distress among Recent Immigrants: A Four-year Longitudinal Study in Israel and Germany. Int Migr (2007) 45(1):151–75. 10.1111/j.1468-2435.2007.00399.x

[8] Plummer DL Slane S Patterns of Coping in Racially Stressful Situations. J Black Psychol (1996) 22(3):302–15. 10.1177/00957984960223002

[9] Heim D Hunter SC Jones R Perceived Discrimination, Identification, Social Capital, and Well-Being. J Cross Cult Psychol (2010) 42(7):1145–64. 10.1177/0022022110383310

[10] Mewes R Asbrock F Laskawi J Perceived discrimination and impaired mental health in Turkish immigrants and their descendents in Germany. Compr Psychiatry (2015) 62:42–50. 10.1016/j.comppsych.2015.06.009 26343466

[11] Clark R Anderson NB Clark VR Williams DR Racism as a stressor for African Americans - A biopsychosocial model. Am Psychol (1999) 54(10):805–16. 10.1037/0003-066x.54.10.805 10540593

[12] Harrell SP A multidimensional conceptualization of racism-related stress: implications for the well-being of people of color. Am J Orthopsychiat (2000) 70(1):42–57. 10.1037/h0087722 10702849

[13] Brondolo E Brady ver Halen N Pencille M Beatty D Contrada RJ Coping with racism: a selective review of the literature and a theoretical and methodological critique. J Behav Med (2009) 32(1):64–88. 10.1007/s10865-008-9193-0 19127420 PMC3258496

[14] Brondolo E Thompson S Brady N Appel R Cassells A Tobin JN The relationship of racism to appraisals and coping in a community sample. Ethn Dis (2005) 15(4 Suppl 5):S5–14-9. 16315377

[15] Park IJK Wang L Williams DR Alegría M Coping With Racism: Moderators of the Discrimination-Adjustment Link Among Mexican-Origin Adolescents. Child Dev (2018) 89(3):e293–310. 10.1111/cdev.12856 PMC6013037 28635029

[16] Dovidio JF Gaertner SE Kawakami K Hodson G Why can’t we just get along? Interpersonal biases and interracial distrust. Cult Divers Ethnic Minor Psychol (2002) 8(2):88–102. 10.1037/1099-9809.8.2.88 11987594

[17] Landrine H Klonoff EA The Schedule of Racist Events: A Measure of Racial Discrimination and a Study of Its Negative Physical and Mental Health Consequences. J Black Psychol (1996) 22(2):144–68. 10.1177/00957984960222002

[18] Sue DW Bucceri J Lin AI Nadal KL Torino GC Racial microaggressions and the Asian American experience. Cult Divers Ethnic Minor Psychol (2007) 13(1):72–81. 10.1037/1099-9809.13.1.72 17227179

[19] Pettigrew TF Meertens RW Subtle and blatant prejudice in western Europe. Eur J Soc Psychol (1995) 25(1):57–75. 10.1002/ejsp.2420250106

[20] Zick A Wagner U van Dick R Petzel T Acculturation and Prejudice in Germany: Majority and Minority Perspectives. J Soc Issues (2001) 57(3):541–57. 10.1111/0022-4537.00228

[21] Sue DW Capodilupo CM Torino GC Bucceri JM Holder AM Nadal KL Racial microaggressions in everyday life: implications for clinical practice. Am Psychol (2007) 62(4):271–86. 10.1037/0003-066X.62.4.271 17516773

[22] Dovidio JF Gaertner SL Aversive racism and selection decisions: 1989 and 1999. Psychol Sci (2000) 11(4):315–9. 10.1111/1467-9280.00262 11273391

[23] Gaertner SL Dovidio JF The aversive form of racism. In: Dovidio JF Gaertner SL, editors. Prejudice, discrimination, and racism. Orlando, FL: Academic Press (1986). p. 61–89.

[24] Jones KP Peddie CI Gilrane VL King EB Gray AL Not So Subtle: A Meta-Analytic Investigation of the Correlates of Subtle and Overt Discrimination. J Manage (2016) 42(6):1588–613. 10.1177/0149206313506466

[25] Noh S Kaspar V Wickrama KA Overt and subtle racial discrimination and mental health: preliminary findings for Korean immigrants. Am J Public Health (2007) 97(7):1269–74. 10.2105/AJPH.2005.085316 PMC1913092 17538066

[26] Cuddy AJ Fiske ST Glick P The BIAS map: behaviors from intergroup affect and stereotypes. J Pers Soc Psychol (2007) 92(4):631–48. 10.1037/0022-3514.92.4.631 17469949

[27] Fiske ST Stereotype Content: Warmth and Competence Endure. Curr Dir Psychol Sci (2018) 27(2):67–73. 10.1177/0963721417738825 29755213 PMC5945217

[28] Fiske ST Cuddy AJC Glick P Xu J. A model of (often mixed) stereotype content: Competence and warmth respectively follow from perceived status and competition. J Pers Soc Psychol (2002) 82(6):878–902. 10.1037/0022-3514.82.6.878 12051578

[29] Ajrouch KJ Reisine S Lim S Sohn W Ismail A Perceived everyday discrimination and psychological distress: does social support matter? Ethn Health (2010) 15(4):417–34. 10.1080/13557858.2010.484050 PMC6436554 20582775

[30] Carlsson M Fumarco L Rooth D-O Ethnic discrimination in hiring, labour market tightness and the business cycle - evidence from field experiments. Appl Econ (2017) 50(24):2652–63. 10.1080/00036846.2017.1406653

[31] Taylor RJ Miller R Mouzon D Keith VM Chatters LM Everyday Discrimination among African American Men: The Impact of Criminal Justice Contact. Race Justice (2018) 8(2):154–77. 10.1177/2153368716661849 PMC5849240 29552376

[32] Auspurg K Schneck A Hinz T Closed doors everywhere? A meta-analysis of field experiments on ethnic discrimination in rental housing markets. J7nbsp;Ethn Migr Stud (2018) 45(1):95–114. 10.1080/1369183x.2018.1489223

[33] Phinney JS Understanding Ethnic Diversity. Am Behav Sci (1996) 40(2):143–52. 10.1177/0002764296040002005

[34] Cameron JE A Three-Factor Model of Social Identity. Self Identity (2004) 3(3):239–62. 10.1080/13576500444000047

[35] Branscombe NR Schmitt MT Harvey RD Perceiving pervasive discrimination among African Americans: Implications for group identification and well-being. J Pers Soc Psychol (1999) 77(1):135–49. 10.1037/0022-3514.77.1.135

[36] Jetten J Haslam SA Cruwys T Branscombe NR Social Identity, Stigma, and Health. In: Major B Dovidio JF Link BG, editors. The Oxford Handbook of Stigma, Discrimination, and Heath. Oxford, UK: Oxford University Press (2017). p. 301–16.

[37] McCoy SK Major B Group identification moderates emotional responses to perceived prejudice. Pers Soc Psychol Bull (2003) 29(8):1005–17. 10.1177/0146167203253466 15189619

[38] Lee DL Ahn S The relation of racial identity, ethnic identity, and racial socialization to discrimination-distress: A meta-analysis of Black Americans. J Couns Psychol (2013) 60(1):1–14. 10.1037/a0031275 23356464

[39] Jasperse M Ward C Jose PE Identity, Perceived Religious Discrimination, and Psychological Well-Being in Muslim Immigrant Women. Appl Psychol (2012) 61(2):250–71. 10.1111/j.1464-0597.2011.00467.x

[40] Bombay A Matheson K Anisman H Decomposing identity: differential relationships between several aspects of ethnic identity and the negative effects of perceived discrimination among First Nations adults in Canada. Cult Divers Ethnic Minor Psychol (2010) 16(4):507–16. 10.1037/a0021373 21058814

[41] Lazarus RS Folkman S Stress, appraisal, and coping. Springer: New York, NY (1984).

[42] Gerrard M Stock ML Roberts ME Gibbons FX O’Hara RE Weng CY Coping with racial discrimination: the role of substance use. Psychol Addict Behav (2012) 26(3):550–60. 10.1037/a0027711 PMC4079542 22545585

[43] Kaholokula JK Antonio MC Ing CK Hermosura A Hall KE Knight R The effects of perceived racism on psychological distress mediated by venting and disengagement coping in Native Hawaiians. BMC Psychol (2017) 5(1):2. 10.1186/s40359-017-0171-6 28081710 PMC5228113

[44] Noh S Kaspar V Perceived discrimination and depression: moderating effects of coping, acculturation, and ethnic support. Am J Public Health (2003) 93(2):232–8. 10.2105/ajph.93.2.232 PMC1447722 12554575

[45] Alvarez AN Juang LP Filipino Americans and racism: A multiple mediation model of coping. J Couns Psychol (2010) 57(2):167–78. 10.1037/a0019091 21133568

[46] Liang CTH Alvarez AN Juang LP Liang MX The role of coping in the relationship between perceived racism and racism-related stress for Asian Americans: Gender differences. J Couns Psychol (2007) 54(2):132–41. 10.1037/0022-0167.54.2.132

[47] Thomas AJ Witherspoon KM Speight SL Gendered racism, psychological distress, and coping styles of African American women. Cult Divers Ethnic Minor Psychol (2008) 14(4):307–14. 10.1037/1099-9809.14.4.307 18954166

[48] Utsey SO Ponterotto JG Reynolds AL Cancelli AA Racial Discrimination, Coping, Life Satisfaction, and Self-Esteem Among African Americans. J Couns Dev (2000) 78(1):72–80. 10.1002/j.1556-6676.2000.tb02562.x

[49] Barnes PW Lightsey OR Perceived Racist Discrimination, Coping, Stress, and Life Satisfaction. J Multicult Couns D (2005) 33(1):48–61. 10.1002/j.2161-1912.2005.tb00004.x

[50] Szymanski DM Obiri O Do Religious Coping Styles Moderate or Mediate the External and Internalized Racism-Distress Links? Couns Psychol (2010) 39(3):438–62. 10.1177/0011000010378895

[51] Krieger N Racial and gender discrimination: Risk factors for high blood pressure? Soc Sci Med (1990) 30(12):1273–81. 10.1016/0277-9536(90)90307-e 2367873

[52] Noh S Beiser M Kaspar V Hou F Rummens J Perceived Racial Discrimination, Depression, and Coping: A Study of Southeast Asian Refugees in Canada. J Health Soc Behav (1999) 40(3):193. 10.2307/2676348 10513144

[53] West LM Donovan RA Roemer L Coping With Racism: What Works and Doesn’t Work for Black Women? J Black Psychol (2009) 36(3):331–49. 10.1177/0095798409353755

[54] Williams DR Yu Y Jackson JS Anderson NB Racial Differences in Physical and Mental Health: Socio-economic Status, Stress and Discrimination. J Health Psychol (1997) 2(3):335–51. 10.1177/135910539700200305 22013026

[55] Komproe IH Rijken M Ros WJG Winnubst JAM tHart H Available Support and Received Support: Different Effects Under Stressful Circumstances. J Soc Pers Relat (1997) 14(1):59–77. 10.1177/0265407597141003

[56] Chataway CJ Berry JW Acculturation experiences, appraisal, coping, and adaptation: A comparison of Hong Kong Chinese, French, and English students in Canada. Can J Beh Sci (1989) 21(3):295–309. 10.1037/h0079820

[57] Davis MH Morris MM Kraus LA Relationship-specific and global perceptions of social support: Associations with well-being and attachment. J Pers Soc Psychol (1998) 74(2):468–81. 10.1037/0022-3514.74.2.468 9491588

[58] Fischer AR Shaw CM African Americans’ mental health and perceptions of racist discrimination: The moderating effects of racial socialization experiences and self-esteem. J Couns Psychol (1999) 46(3):395–407. 10.1037/0022-0167.46.3.395

[59] Sanders Thompson VL Coping Responses and the Experience of Discrimination. J Appl Soc Psychol (2006) 36(5):1198–214. 10.1111/j.0021-9029.2006.00038.x

[60] Vega W Kolody B Valle R Weir J Social Networks, Social Support, and their Relationship to Depression among Immigrant Mexican Women. Hum Organ (1991) 50(2):154–62. 10.17730/humo.50.2.p340266397214724

[61] Krieger N Sidney S Racial discrimination and blood pressure: the CARDIA Study of young black and white adults. Am J Public Health (1996) 86(10):1370–8. 10.2105/ajph.86.10.1370 PMC1380646 8876504

[62] Kim IH Noh S Racial/ethnic variations in the main and buffering effects of ethnic and nonethnic supports on depressive symptoms among five ethnic immigrant groups in Toronto. Ethn Health (2016) 21(3):215–32. 10.1080/13557858.2015.1061101 26159597

[63] Clark R Perceived racism and vascular reactivity in black college women: moderating effects of seeking social support. Health Psychol (2006) 25(1):20–5. 10.1037/0278-6133.25.1.20 16448294

[64] Ivakhnyuk I The russian migration policy and its impact on human development: The historical perspective. United Nations Development Programme: New York, NY (2009). Available at: https://mpra.ub.uni-muenchen.de/19196/.

[65] Federal Statistical Office of Germany Bevölkerung mit Migrationshintergrund – Ergebnisse des Mikrozensus 2017. Wiesbaden, Germany: Federal Statistical Office of Germany (2019). Available at: https://www.destatis.de/DE/Themen/Gesellschaft-Umwelt/Bevoelkerung/Migration-Integration/Publikationen/Downloads-Migration/migrationshintergrund-2010220187004.pdf.

[66] Tselmin S Korenblum W Reimann M Bornstein SR Schwarz PE The health status of Russian-speaking immigrants in Germany. Horm Metab Res (2007) 39(12):858–61. 10.1055/s-2007-993153 18075968

[67] Dietz B German and Jewish migration from the former Soviet Union to Germany: Background, trends and implications. J Ethn Migr Stud (2000) 26(4):635–52. 10.1080/713680499

[68] Salentin K Determinants of Experience of Discrimination in Minorities in Germany. Int J Confl Violence (2007) 1(1):32–50. 10.4119/UNIBI/ijcv.19

[69] Titzmann PF Silbereisen RK Mesch GS Schmitt-Rodermund E Migration-Specific Hassles Among Adolescent Immigrants From the Former Soviet Union in Germany and Israel. J Cross Cult Psychol (2011) 42(5):777–94. 10.1177/0022022110362756

[70] Aparicio ML Döring A Mielck A Holle R Unterschiede zwischen Aussiedlern und der übrigen deutschen Bevölkerung bezüglich Gesundheit, Gesundheitsversorgung und Gesundheitsverhalten: eine vergleichende Analyse anhand des KORA-Surveys 2000. Soz Praventivmed (2005) 50(2):107–18. 10.1007/s00038-004-3088-9 15900963

[71] Deckert A Winkler V Meisinger C Heier M Becher H Myocardial infarction incidence and ischemic heart disease mortality: overall and trend results in repatriates, Germany. Eur J Public Health (2014) 24(1):127–33. 10.1093/eurpub/ckt058 23729483

[72] Kuhrs E Winkler V Becher H Risk factors for cardiovascular and cerebrovascular diseases among ethnic Germans from the former Soviet Union: results of a nested case-control study. BMC Public Health (2012) 12:190. 10.1186/1471-2458-12-190 22413759 PMC3317863

[73] Volodina A Bertsche T Kostev K Winkler V Haefeli WE Becher H Drug utilization patterns and reported health status in ethnic German migrants (Aussiedler) in Germany: a cross-sectional study. BMC Public Health (2011) 11:509. 10.1186/1471-2458-11-509 21711531 PMC3141468

[74] Rask S Suvisaari J Koskinen S Koponen P Molsa M Lehtisalo R The ethnic gap in mental health: A population-based study of Russian, Somali and Kurdish origin migrants in Finland. Scand J Public Health (2016) 44(3):281–90. 10.1177/1403494815619256 26647096

[75] Krieger N Discrimination and Health. In: Berkman LF Kawachi I M GM, editors. Social Epidemiology. Oxford, England: Oxford University Press (2014). p. 63–125.

[76] Barwick C Draußen vor der Tür: Exklusion auf dem Berliner Wohnungsmarkt. WZB Mitt (2011) 134:13–5.

[77] Ababkov VA Barisnikov K Vorontzova-Wenger OV Gorbunov IA Kapranova SV Pologaeva EA Validation of the Russian version of the questionnaire “Scale of perceived stress–10”. Vestnik Saint Petersburg Univ (2016) 16(2):6–15. 10.21638/11701/spbu16.2016.202

[78] Sibley CG The BIAS-Treatment Scale (BIAS-TS): a measure of the subjective experience of active and passive harm and facilitation. J Pers Assess (2011) 93(3):300–15. 10.1080/00223891.2011.559389 21516589

[79] Rosseel Y lavaan: An R Package for Structural Equation Modeling. J Stat Softw (2012) 48(2):1–36. 10.18637/jss.v048.i02

[80] Forman TA Williams DR Jackson JS Race, Place, and Discrimination. In: Gardner C, editor. Perspectives on Social Problems. Greenwich, CT: JAI Press (1997). p. 231–61.

[81] Beigang S Fetz K Kalkum D Otto M Diskriminierungserfahrungen in Deutschland. Baden-Baden, Germany: Nomos (2017).

[82] Cohen S Kamarck T Mermelstein R. A Global Measure of Perceived Stress. J Health Soc Behav (1983) 24(4):385–96. 10.2307/2136404 6668417

[83] Cohen S Williamson GM Perceived stress in a probability sample in the United States. In: Spacapan S Oskamp S, editors. The Social Psychology of Health. Newbury Park, CA: Sage (1988). p. 31–67.

[84] Simon B Grabow O The Politicization of Migrants: Further Evidence that Politicized Collective Identity is a Dual Identity. Polit Psychol (2010) 31(5):717–38. 10.1111/j.1467-9221.2010.00782.x

[85] Simon B Ruhs D Identity and politicization among Turkish migrants in Germany: the role of dual identification. J Pers Soc Psychol (2008) 95(6):1354–66. 10.1037/a0012630 19025288

[86] Carver CS You want to measure coping but your protocol’s too long: consider the brief COPE. Int J Behav Med (1997) 4(1):92–100. 10.1207/s15327558ijbm0401_6 16250744

[87] Carver CS Scheier MF Weintraub JK Assessing coping strategies: A theoretically based approach. J Pers Soc Psychol (1989) 56(2):267–83. 10.1037/0022-3514.56.2.267 2926629

[88] Heller K Swindle RW Social networks, perceived social support, and coping with stress. In: Felner RD Jason LA Moritsugu JN Farber SS, editors. Preventive Psychology: Theory, Research and Practice. New York, NY: Pergamon (1983). p. 87–103.

[89] Norberg AL Lindblad F Boman KK Support-seeking, perceived support, and anxiety in mothers and fathers after children’s cancer treatment. Psychooncology (2006) 15(4):335–43. 10.1002/pon.960 16106491

[90] Ognibene TC Collins NL Adult Attachment Styles, Perceived Social Support and Coping Strategies. J Soc Pers Relat (1998) 15(3):323–45. 10.1177/0265407598153002

[91] Brown TL Phillips CM Abdullah T Vinson E Robertson J Dispositional Versus Situational Coping: Are the Coping Strategies African Americans Use Different for General Versus Racism-Related Stressors? J Black Psychol (2010) 37(3):311–35. 10.1177/0095798410390688

[92] Knoll N Rieckmann N Schwarzer R Coping as a mediator between personality and stress outcomes: a longitudinal study with cataract surgery patients. Eur J Pers (2005) 19(3):229–47. 10.1002/per.546

[93] Mitchell PH Powell L Blumenthal J Norten J Ironson G Pitula CR A short social support measure for patients recovering from myocardial infarction: the ENRICHD Social Support Inventory. J Cardiopulm Rehabil (2003) 23(6):398–403. 10.1097/00008483-200311000-00001 14646785

[94] Kendel F Spaderna H Sieverding M Dunkel A Lehmkuhl E Hetzer R Eine deutsche Adaptation des ENRICHD Social Support Inventory (ESSI). Diagnostica (2011) 57(2):99–106. 10.1026/0012-1924/a000030

[95] Ruggiero KM Taylor DM Lydon JE How Disadvantaged Group Members Cope With Discrimination When They Perceive That Social Support Is Available. J Appl Soc Psychol (1997) 27(18):1581–600. 10.1111/j.1559-1816.1997.tb01614.x

[96] Swiss Centre of Expertise in the Social Sciences Swiss Household Panel (n. d.) [2019-03-11]. Lausanne, Switzerland: Swiss Centre of Expertise in the Social Sciences (2011). Available at: https://forscenter.ch/projects/swiss-household-panel/.

[97] Hayes AF PROCESS: A versatile computational tool for observed variable mediation, moderation, and conditional process modeling. (2012). Available at: http://www.afhayes.com/public/process2012.pdf.

[98] Aiken LS West SG Multiple regression: Testing and interpreting interactions. Sage: Newbury Park, CA (1991).

[99] Preacher KJ Hayes AF Asymptotic and resampling strategies for assessing and comparing indirect effects in multiple mediator models. Behav Res Methods (2008) 40(3):879–91. 10.3758/brm.40.3.879 18697684

[100] Cohen J Statistical power analysis for the behavioral sciences. 2nd ed Routledge: Hillsdale, NJ (1988).

[101] Torres L Taknint JT Ethnic microaggressions, traumatic stress symptoms, and Latino depression: A moderated mediational model. J Couns Psychol (2015) 62(3):393–401. 10.1037/cou0000077 25867692

[102] Krings F Johnston C Binggeli S Maggiori C Selective incivility: immigrant groups experience subtle workplace discrimination at different rates. Cult Divers Ethnic Minor Psychol (2014) 20(4):491–8. 10.1037/a0035436 25133409

[103] Stepanikova I Oates GR Perceived Discrimination and Privilege in Health Care: The Role of Socioeconomic Status and Race. Am J Prev Med (2017) 52(1S1):S86–94. 10.1016/j.amepre.2016.09.024 PMC5172593 27989297

[104] Zschirnt E Ruedin D Ethnic discrimination in hiring decisions: a meta-analysis of correspondence tests 1990–2015. J Ethn Migr Stud (2016) 42(7):1115–34. 10.1080/1369183x.2015.1133279

[105] Ong AD Fuller-Rowell T Burrow AL Racial discrimination and the stress process. J Pers Soc Psychol (2009) 96(6):1259–71. 10.1037/a0015335 19469600

[106] Fuller-Rowell TE Ong AD Phinney JS National Identity and Perceived Discrimination Predict Changes in Ethnic Identity Commitment: Evidence from a Longitudinal Study of Latino College Students. Appl Psychol (2013) 62(3):406–26. 10.1111/j.1464-0597.2012.00486.x

[107] Operario D Fiske ST Ethnic Identity Moderates Perceptions of Prejudice: Judgments of Personal Versus Group Discrimination and Subtle Versus Blatant Bias. Pers Soc Psychol B (2016) 27(5):550–61. 10.1177/0146167201275004

[108] Outten HR Schmitt MT Garcia DM Branscombe NR Coping Options: Missing Links between Minority Group Identification and Psychological Well-Being. Appl Psychol (2008) 58(1):146–70. 10.1111/j.1464-0597.2008.00386.x

[109] Sellers RM Shelton JN The role of racial identity in perceived racial discrimination. J Pers Soc Psychol (2003) 84(5):1079–92. 10.1037/0022-3514.84.5.1079 12757150

[110] Skrobanek J Perceived Discrimination, Ethnic Identity and the (Re-) Ethnicisation of Youth with a Turkish Ethnic Background in Germany. J Ethn Migr Stud (2009) 35(4):535–54. 10.1080/13691830902765020

[111] Quintana SM Racial and ethnic identity: Developmental perspectives and research. J Couns Psychol (2007) 54(3):259–70. 10.1037/0022-0167.54.3.259

[112] Doyle DM Molix L Perceived discrimination as a stressor for close relationships: identifying psychological and physiological pathways. J Behav Med (2014) 37(6):1134–44. 10.1007/s10865-014-9563-8 24659156

[113] Tice DM Bratslavsky E Giving in to Feel Good: The Place of Emotion Regulation in the Context of General Self-Control. Psychol Inq (2000) 11(3):149–59. 10.1207/s15327965pli1103_03

[114] Tull ES Sheu YT Butler C Cornelious K Relationships between Perceived Stress, Coping Behavior and Cortisol Secretion in Women with High and Low Levels of Internalized Racism. J Natl Med Assoc (2005) 97:206–12. PMC2568780 15712783

[115] Martin RC Dahlen ER Cognitive emotion regulation in the prediction of depression, anxiety, stress, and anger. Pers Individ Dif (2005) 39(7):1249–60. 10.1016/j.paid.2005.06.004

[116] Prelow HM Mosher CE Bowman MA Perceived racial discrimination, social support, and psychological adjustment among African American college students. J Black Psychol (2006) 32(4):442–54. 10.1177/0095798406292677

[117] Jasinskaja-Lahti I Liebkind K Jaakkola M Reuter A Perceived Discrimination, Social Support Networks, and Psychological Well-being Among Three Immigrant Groups. J Cross Cult Psychol (2016) 37(3):293–311. 10.1177/0022022106286925

[118] Perez SM Gavin JK Diaz VA Stressors and coping mechanisms associated with perceived stress in Latinos. Ethn Dis (2015) 25(1):78–82. 25812256

[119] Wei M Ku TY Russell DW Mallinckrodt B Liao KY Moderating effects of three coping strategies and self-esteem on perceived discrimination and depressive symptoms: A minority stress model for Asian international students. J Couns Psychol (2008) 55(4):451–62. 10.1037/a0012511 22017552

[120] Farley T Galves A Dickinson LM Perez MD Stress, coping, and health: a comparison of Mexican immigrants, Mexican-Americans, and non-Hispanic whites. J Immigr Health (2005) 7(3):213–20. 10.1007/s10903-005-3678-5 15900422

[121] Goldbeck R Denial in physical illness. J Psychosom Res (1997) 43(6):575–93. 10.1016/s0022-3999(97)00168-2 9430071

[122] Lazarus RS The costs and benefits of denial. In: Breznitz S, editor. The Denial of Stress. New York, NY: International Universities Press (1983). p. 3–30.

[123] Hoggard LS Byrd CM Sellers RM Comparison of African American college students’ coping with racially and nonracially stressful events. Cult Divers Ethnic Minor Psychol (2012) 18(4):329–39. 10.1037/a0029437 22866688

[124] Raphael KG Cloitre M Does mood-congruence or causal search govern recall bias? a test of life event recall. J Clin Epidemiol (1994) 47(5):555–64. 10.1016/0895-4356(94)90302-6 7730881

[125] Adam EK Heissel JA Zeiders KH Richeson JA Ross EC Ehrlich KB Developmental histories of perceived racial discrimination and diurnal cortisol profiles in adulthood: A 20-year prospective study. Psychoneuroendocrinology (2015) 62:279–91. 10.1016/j.psyneuen.2015.08.018 PMC4739843 26352481

