# Supplementary material for: What Mediates the Relationship Between Ethnic Discrimination and Stress? Coping Strategies and Perceived Social Support of Russian Immigrants in Germany
Source: Front Psychiatry. 2020 Sep 15;11:557148. doi: 10.3389/fpsyt.2020.557148 (PMC7533615; doi:10.3389/fpsyt.2020.557148)
Supplement: Supplementary file 1 [file Table_1.docx]

Supplementary Material

**Supplementary Table 1.** Coefficients and indirect effects for the three models testing the influence of different forms of discrimination on perceived stress mediated by individual coping and social support.

|  | **Model 1: Active Harm** | | | | **Model 2: Passive Harm** | | | | **Model 3: Everyday Discrimination** | | | |
| --- | --- | --- | --- | --- | --- | --- | --- | --- | --- | --- | --- | --- |
|  | Path a: To Coping/Social Support | Path b:  To Stress | Indirect Effect (Boot CI) | *SE* | Path a: To Coping/Social Support | Path b:  To Stress | Indirect Effect (Boot CI) | *SE* | Path a: To Coping/Social Support | Path b:  To Stress | Indirect Effect (Boot CI) | *SE* |
| **Full Model** |  |  |  |  |  |  |  |  |  |  |  |  |
| Active Coping | 0.10 | 0.009 | 0.001  (-0.01, 0.02) | 0.007 | 0.17*** | -0.009 | -0.002  (-0.02, 0.02) | 0.01 | 0.25*** | -0.01 | -0.003  (-0.03, 0.03) | 0.02 |
| Substance Use | 0.09 | 0.27*** | 0.03  (-0.001, 0.070) | 0.02 | 0.11*** | 0.25*** | **0.03**  **(0.01, 0.05)** | 0.01 | 0.07* | 0.27*** | 0.02  (-0.001, 0.04) | 0.01 |
| Venting | 0.14 | 0.08 | 0.01  (-0.002, 0.04) | 0.009 | 0.08* | 0.09* | 0.007  (-0.001, 0.02) | 0.005 | 0.17*** | 0.08 | **0.01**  **(0.001, 0.03)** | 0.008 |
| Humor & Positive Reframing | 0.04 | 0.004 | 0.001  (-0.006, 0.01) | 0.004 | 0.06 | 0.00 | 0.001  (-0.008, 0.009) | 0.004 | 0.10* | 0.01 | 0.001  (-0.009, 0.01) | 0.006 |
| Behavioral Disengagement | 0.06 | 0.11* | 0.006  (-0.005, 0.03) | 0.008 | 0.06 | 0.11* | 0.006  (-0.001, 0.02) | 0.005 | 0.14** | 0.10* | **0.01**  **(0.003, 0.03)** | 0.008 |
| Religion | 0.29*** | -0.06 | -0.02  (-0.05, 0.007) | 0.01 | 0.12** | -0.05 | -0.006  (-0.02, 0.002) | 0.006 | 0.16** | -0.06 | -0.009  (-0.02, 0.002) | 0.007 |
| Denial | 0.11 | -0.10* | -0.01  (-0.04, 0.001) | 0.001 | 0.10* | -0.10* | -0.01  (-0.03, 0.001) | 0.006 | 0.15** | -0.10* | **-0.02**  **(-0.03, -0.003)** | 0.009 |
| Self-Blame | 0.14 | 0.14** | **0.02**  **(0.005, 0.05)** | 0.01 | 0.20*** | 0.12** | **0.03**  **(0.008, 0.05)** | 0.01 | 0.19*** | 0.14** | **0.03**  **(0.01, 0.04)** | 0.01 |
| Social Support | 0.01 | -0.16*** | -0.002  (-0.03, 0.02) | 0.01 | -0.04 | -0.16*** | 0.006  (-0.005, 0.02) | 0.007 | -0.10* | -0.15*** | **0.02**  **(0.003, 0.04)** | 0.01 |
| **Final model** |  |  |  |  |  |  |  |  |  |  |  |  |
| Substance Use | **–** | **–** | – | – | – | 0.24*** | **0.03**  **(0.01, 0.05)** | 0.01 | – | – | **–** | – |
| Venting | **–** | **–** | – | – | – | – | – | – | – | 0.11** | **0.02 (0.006, 0.04)** | 0.009 |
| Behavioral Disengagement | **–** | **–** | – | – | – | – | – | – | – | 0.10* | **0.02**  **(0.004, 0.03)** | 0.008 |
| Denial | **–** | **–** | – | – | – | – | – | – | – | -0.09* | **-0.01**  **(-0.03, -0.002)** | 0.008 |
| Self-Blame | **–** | 0.21*** | **0.03**  **(0.003, 0.08)** | 0.02 | – | 0.15*** | **0.03**  **(0.01, 0.06)** | 0.01 | – | 0.15** | **0.03**  **(0.01, 0.05)** | 0.01 |
| Social Support | **–** | **–** | – | – | – | – | – | – | – | -0.15*** | **0.02 (0.003, 0.03)** | 0.009 |

*Note.* * *p* < .05, ** *p* < .01, *** *p* < .001. Path a = regression of the form of discrimination on potential mediators. Path b = regression of each potential mediator on stress, controlling for all other mediators. Indirect effects in the final model are computed after excluding irrelevant potential mediators (i.e., bootstrapped confidence intervals included zero) from the models. Indirect effects with confidence intervals entirely above or below zero are depicted in bold. Control variables: age, sex. Confidence intervals are bootstrapped based on 10,000 samples.
